# Gait Variability Is Associated With Gray Matter Volumes Implicated in Cognitive Function: A Cross-Sectional Analysis From the AGUEDA Trial

**DOI:** 10.1093/geroni/igaf045

**Published:** 2025-05-06

**Authors:** Isabel Martín-Fuentes, Patricio Solis-Urra, Emilio J Ruiz-Malagón, Andrea Coca-Pulido, Angel Toval, Beatriz Fernandez-Gamez, Marcos Olvera-Rojas, Darío Bellón, Alessandro Sclafani, Jose Mora-Gonzalez, Lucía Sánchez-Aranda, Javier Sanchez-Martinez, José Pablo Martínez-Barbero, Manuel Gómez-Río, Teresa Liu-Ambrose, Kirk I Erickson, Francisco B Ortega, Irene Esteban-Cornejo

**Affiliations:** Faculty of Sport Sciences, Department of Physical Education and Sports, Sport and Health University Research Institute (iMUDS), University of Granada, Granada, Spain; AdventHealth Research Institute, Neuroscience, Orlando, Florida, USA; Faculty of Education and Social Sciences, Universidad Andres Bello, Viña del Mar, Chile; Faculty of Sport Sciences, Department of Physical Education and Sports, Sport and Health University Research Institute (iMUDS), University of Granada, Granada, Spain; Faculty of Sport Sciences, Department of Physical Education and Sports, Sport and Health University Research Institute (iMUDS), University of Granada, Granada, Spain; Faculty of Sport Sciences, Department of Physical Education and Sports, Sport and Health University Research Institute (iMUDS), University of Granada, Granada, Spain; Faculty of Sport Sciences, Department of Physical Education and Sports, Sport and Health University Research Institute (iMUDS), University of Granada, Granada, Spain; Faculty of Sport Sciences, Department of Physical Education and Sports, Sport and Health University Research Institute (iMUDS), University of Granada, Granada, Spain; Faculty of Sport Sciences, Department of Physical Education and Sports, Sport and Health University Research Institute (iMUDS), University of Granada, Granada, Spain; Faculty of Sport Sciences, Department of Physical Education and Sports, Sport and Health University Research Institute (iMUDS), University of Granada, Granada, Spain; Faculty of Sport Sciences, Department of Physical Education and Sports, Sport and Health University Research Institute (iMUDS), University of Granada, Granada, Spain; Faculty of Sport Sciences, Department of Physical Education and Sports, Sport and Health University Research Institute (iMUDS), University of Granada, Granada, Spain; Faculty of Sport Sciences, Department of Physical Education and Sports, Sport and Health University Research Institute (iMUDS), University of Granada, Granada, Spain; Nuclear Medicine Services, Servicio de Radiodiagnóstico, Hospital Universitario Virgen de las Nieves, Granada, Spain; Instituto de Investigación Biosanitaria ibs. GRANADA, Granada, Spain; Nuclear Medicine Services, Servicio de Radiodiagnóstico, Hospital Universitario Virgen de las Nieves, Granada, Spain; Instituto de Investigación Biosanitaria ibs. GRANADA, Granada, Spain; Faculty of Medicine, Department of Physical Therapy, University of British Columbia, Vancouver, Canada; Centre for Aging SMART at Vancouver Coastal Health, Vancouver Coastal Health Research Institute, British Columbia, Vancouver, Canada; AdventHealth Research Institute, Neuroscience, Orlando, Florida, USA; Faculty of Sport Sciences, Department of Physical Education and Sports, Sport and Health University Research Institute (iMUDS), University of Granada, Granada, Spain; Centro de Investigación Biomédica en Red Fisiopatología de la Obesidad y Nutrición (CIBEROBN), Instituto de Salud Carlos III, Madrid, España; Faculty of Sport Sciences, Department of Physical Education and Sports, Sport and Health University Research Institute (iMUDS), University of Granada, Granada, Spain; Instituto de Investigación Biosanitaria ibs. GRANADA, Granada, Spain; Centro de Investigación Biomédica en Red Fisiopatología de la Obesidad y Nutrición (CIBEROBN), Instituto de Salud Carlos III, Madrid, España

**Keywords:** Brain volumes, Brain structure, Gait inconsistency, Magnetic resonance imaging

## Abstract

**Background and Objectives:**

Aging is associated with both gait impairments and cognitive decline; however, the relationship between specific gait variability parameters, gray matter volume (GMV), and cognitive function remains poorly understood. This study aims to examine the associations between gait variability parameters (derived from stride length, step length, step time, and gait velocity) and GMV and its associations with cognitive function in cognitively normal older adults.

**Research Design and Methods:**

Eighty-seven older adults (48 female) aged 65–80 from the AGUEDA trial participated in this cross-sectional analysis. The Optogait system was used to record gait parameters. T1-weighted brain images were acquired magnetic resonance imaging scanner, and GMV was calculated by whole-brain voxel-based morphometric analysis using SPM12. Cognitive function was calculated from different cognitive tests.

**Results:**

Greater stride length variability was associated with lower GMV (*p* < .001) in clusters located in the supramarginal gyrus (*t* = 4.014, *k* = 179, β = -0.494) and hippocampus (*t* = 3.670, *k* = 334, β = -0.394), whereas greater step length variability was linked to lower GMV in the parahippocampal gyrus (*t* = 3.624, *k* = 76, β = -0.410). However, greater step time variability was associated with greater GMV in the supplementary motor area (*t* = 4.117, *k* = 274, β = 0.449). Gait velocity variability did not show any association with GMV. Furthermore, greater GMV in the supramarginal gyrus was associated with better working memory (β = 0.252, *p* = .008); greater GMV in the hippocampus was associated with better attentional/inhibitory control (β = 0.275, *p* = .010); and greater GMV in the parahippocampal gyrus was associated with better EF (β = 0.212, *p* = .035), attentional/inhibitory control (β = 0.241, *p* = .019), and working memory (β = 0.233, *p* = .027).

**Discussion and Implications:**

These results suggest that gait variability could be an indicator of neurocognitive decline in older adults. Understanding these associations is essential for early dementia detection and sheds light on the complex interplay between physical function, brain health, and cognitive function during aging.

**Translational Significance:** Aging is associated with gait impairments and cognitive decline, yet the relationship between gait variability parameters, gray matter volume (GMV), and cognitive function is not well understood. This study revealed associations between gait variability parameters and GMV in regions such as supramarginal gyrus, hippocampus, and parahippocampal gyrus, which were also linked to cognitive function. These findings highlight gait variability as a potential biomarker for early neurocognitive decline. Translation into practice could include gait-based screening tools, interventions to mitigate cognitive decline, and policies promoting physical and cognitive health monitoring.

## Background and Objectives

Aging affects neurocognitive and physical capacities, but the interdependent nature of these associations remains a source of discussion and debate (Cullen et al., 2018; Erickson et al., 2022; Kramer & Erickson, 2007). For instance, age-related changes in gait begin earlier than detectable changes in cognition, and may precede and predict the development of dementia (Ashburner & Friston, 2000; Beauchet et al., 2009). Specifically, gait variability (i.e., the stride-to-stride fluctuations during gait) has been established as a sensitive early noninvasive marker for brain atrophy and cognitive decline (Montero-Odasso et al., 2020).

Associations between gait parameters, brain outcomes, and their associations with cognitive decline have become more firmly established (Cullen et al., 2018; Montero-Odasso et al., 2020). Particularly, greater gait variability is considered an index of poor gait stability associated with an increased risk of falls, fractures, and frailty in older adults (Cullen et al., 2018). Also, greater gait variability is associated with reduced gray matter volume (GMV) in older adults (Cosentino et al., 2020; Rosso et al., 2014; Skillbäck et al., 2022). For instance, greater gait variability was associated with reduced GMV in hippocampal and medial temporal lobe regions in older adults with mild cognitive impairment (Cosentino et al., 2020; Li et al., 2016). Moreover, greater stride length variability was associated with reduced GMV in the left lingual region (Ali et al., 2022), and greater step length variability was associated with hippocampus (Jayakody et al., 2021; Rosso et al., 2014) and anterior cingulate cortex atrophy in healthy older adults (Jayakody et al., 2020; Rosso et al., 2014). In contrast, greater step length variability was associated with greater GMV in frontal, left angular parietal, superior, and middle inferior temporal regions in healthy older adults. This finding suggests that the direction of these associations may depend on the specific brain regions involved, enhancing our understanding of the shared cerebral networks that may underlie individual differences in gait variability and cognitive functions in older adults (Jayakody et al., 2021).

Increases in gait variability have been shown through all states, from early mild cognitive impairment to more advanced stages of dementia and neurodegenerative diseases (Herssens et al., 2020; Kueper et al., 2017; Skillbäck et al., 2022). Most of the previous literature has shown associations between gait variability and GMV in neurodegenerative conditions and aging. However, there are different approaches in the protocols, such as using different brain measurement techniques (Rosso et al., 2014), evaluating only specific regions of the brain (Annweiler et al., 2013; Beauchet et al., 2015; Jayakody et al., 2020; Zimmerman et al., 2009), including cognitive impaired participants (Annweiler et al., 2013; Lipat et al., 2022), or including relatively small sample sizes (Ali et al., 2022). Addressing these methodological variations is essential to fully understand the relationships between gait variability, GMV, and cognitive function in older adults. To this extent, the evidence presented is inconsistent, the most of the evidence arise from specific regions of interest analysis, and a comprehensive exploration of these associations through a whole-brain approach and extending to an assessment of its cognitive implications in cognitively normal older adults is needed (Li et al., 2016; Montero-Odasso et al., 2020). This is crucial to gain insight into the gait variability changes as potential early indicators of cognitive decline.

Likewise, poor cognitive function (Diamond, 2013), which directly affects activities of daily living (Pieruccini-Faria et al., 2020), typically coexists with motor and gait alterations (Herman et al., 2010; Pieruccini-Faria et al., 2020; Van Iersel et al., 2008). Indeed, greater gait variability was associated with poor cognitive function in normal aging (Herman et al., 2010; Van Iersel et al., 2008), as well as in people with neurodegenerative disorders (Muir et al., 2012; Pieruccini-Faria et al., 2020). For example, both greater stride length and stride time variability have been identified as markers of poorer executive function (EF; Hausdorff et al., 2001; Ijmker & Lamoth, 2012; Párraga-Montilla et al., 2021). Despite this, the associations between gait variability and its coupling with GMV and cognitive function require further investigation (Pieruccini-Faria et al., 2020; Sukits et al., 2014). In this study, we strive to provide a comprehensive exploration of these associations through a whole-brain approach, integrating assessments of gait variability, GMV, and cognitive function in cognitively normal older adults. By doing so, we seek to offer a novel perspective on the interplay between motor, brain, and cognitive functions, which may reveal early indicators of cognitive decline and contribute uniquely to the existing literature.

Therefore, the aim of this study was twofold: (i) to examine the associations between gait variability parameters (derived by stride length, step length, step time, and gait velocity) and GMV using a voxel-wise whole-brain approach, and (ii) to investigate whether gait variability-related GMV brain regions are associated with cognitive function in a sample of cognitively normal older adults.

## Research Design and Methods

### Participants

Baseline data from 91 cognitively normal older adults aged 65–80 years (52 female) who participated in the Active Gains in brain Using Exercise During Aging (AGUEDA) trial were considered for this cross-sectional analysis. Participants were recruited from Granada (Spain). All were defined as physically inactive and cognitively normal according to the Spanish Telephone Interview for Cognitive Status modified (score ≥26/41; Muñoz-García et al., 2019), the Mini-Mental State Examination (≥25/30; Blesa et al., 2001) and the Montreal Cognitive Assessment (cutoﬀs according to age; <71 years, ≥24/30; 71–75, ≥ 22/30; >75, 21/30; and adjusted by years of education for a Spanish population; Ojeda et al., 2016). Further description of the eligibility criteria, sources, and methods of participants’ selection, and information regarding setting, locations, recruitment, and data collection have been previously described (Fernandez-Gamez et al., 2023; Solis-Urra et al., 2023). Informed consent was obtained for experimentation with human subjects. Participants signed an informed consent prior to enrolling in the AGUEDA trial, which was approved by the Research Ethics Board of the Andalusian Health Service (CEIM/CEI Provincial de Granada; #2317-N-19) on May 25, 2020. The AGUEDA trial is registered in ClinicalTrials.gov (identifier: NCT05186090).

### Gait Variability Parameters

**Figure 1.**
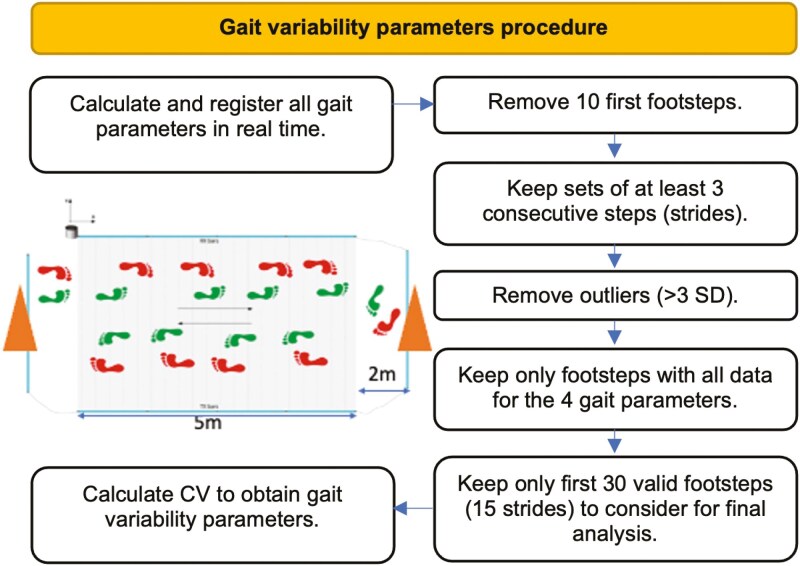
Flow diagram of the procedure followed to obtain gait variability parameters. CV = coefficient of variation; *SD* = standard deviation.

The Optogait system (Microgate Slr; Bolzano, Italy) was used to determine gait variability parameters (stride length, step length, step time, and gait velocity; Lee, Song, Lee, Jung, et al., 2014; Lee, Song, Lee, Shin, et al., 2014). Participants walked back and forth across a track made up of two bars (i.e., a transmitting and a receiving optical signal bar) set 1 m apart at the maximum walking pace that they could maintain for 5 min, recording a minimum of 150 steps. The bars were 5 m long (5 segments of 1 m each), and two cones were placed 2 m apart at each end of the track, so the participants had to turn around the cone and walk through the track again (Figure 1). Each single transmitter bar contains 96 infrared LEDs (1,041 cm resolution), which are continuously communicating with the LEDs on the receiver bar. The system measures all parameters with an accuracy of 1/1,000 of a second (Párraga-Montilla et al., 2021).

The software calculated and registered all gait parameters in real time (see Supplementary Figure S1). The first 10 footsteps were removed from the analysis to avoid unsteady gait data and sets of at least three consecutive steps were selected to certify that only actual strides were chosen (Hausdorff et al., 2001). Next, outliers (>3*SD*) were removed for each parameter (Hausdorff et al., 2001), and we kept only footsteps with all data for the four gait parameters (see Supplementary Table S1 for descriptive characteristics of gait parameters). Only the first 30 valid footsteps (15 strides) of each participant were considered for further analysis (Kroneberg et al., 2019). Afterwards, the coefficient of variation (CV) of stride length (cm), step length (cm), step time (s), and gait velocity (m/s) were calculated as gait variability parameters (Montero-Odasso et al., 2019; Figure 1).

### Magnetic Resonance Imaging Procedure

#### Data acquisition

Brain images were acquired on a 3.0 T Siemens Magnetom PRISMA Fit scanner with a 64-channel head coil (Siemens Medical Solutions, Erlangen, Germany). A magnetization-prepared rapid gradient-echo (MPRAGE) sequence was used to collect the three-dimensional, high-resolution, T1-weighted images. The acquisition parameters were as follows: repetition time (TR) = 2,400 ms, echo time (TE) = 2.31 ms, inversion time (IT) = 1,060 ms, field of view (FOV) = 256 mm, resolution = 0.8 × 0.8 × 0.8 mm, 224 slices, and scan duration of 6 min and 38 s.

#### Structural image processing

Statistical Parametric Mapping software (SPM12; Wellcome Department of Cognitive Neurology, MA, USA) implemented in Matlab (The MathWorks, Inc., Natick, MA, USA) was used to preprocess structural imaging data. Two independent reviewers separately checked each individual image for quality control and identification of acquisition artifacts. No images were excluded based on image quality.

Preprocessing steps were as follows. First, T1-weighted structural images were segmented into gray matter tissue, white matter tissue, and cerebrospinal fluid (Ashburner & Friston, 2005) using the segmentation algorithm implemented in SPM12. Second, a customized template using Diffeomorphic Anatomical Registration Through Exponentiated Lie Algebra (DARTEL) was created using the segmented gray matter/white matter tissues (Ashburner, 2007). Then, DARTEL estimated the best set of smooth deformations for each participant’s tissue to their common average, and this process was repeated until convergence. Afterwards, the resultant images were spatially normalized to Montreal Neurological Institute (MNI) space with an affine transformation to create the DARTEL template via nonlinear transformation. The normalized gray matter images were modulated with Jacobian determinants derived from the spatial normalization to perform a volume change correction (Ashburner & Friston, 2000). Finally, the volumetric images were smoothed by convolving them with an isotropic Gaussian kernel of 8 mm full width at half-maximum.

### Cognitive Function: EF Composite Score and Cognitive Domains

An EF composite score was calculated by conducting a confirmatory factor analysis (Coca‐Pulido et al., 2024). All raw scores from the cognitive tests were converted to *z*-scores. The tests included in the EF composite score were the Trail Making Test (TMT), the Digit Symbol Substitution Test (DSST), the Dimensional Change Card Sort Test (DCCS), and the Spatial Working Memory test.

Cognitive domains evaluated were attentional/inhibitory control, episodic memory, processing speed, visuospatial memory, and working memory (Oberlin et al., 2024). Each cognitive domain included scores of different cognitive tests. Supplementary Table S2 provides detailed information on the variables included in each cognitive domain. Extended information and references about the cognitive tests were reported elsewhere (Solis-Urra et al., 2023).

### Confounders

Age, sex, and years of education (the number of years of formal education that each participant completed) were included as potential confounders (Beauchet et al., 2015; Cosentino et al., 2020; Erickson et al., 2022; Jayakody et al., 2021). Body mass index (BMI; Ijmker & Lamoth, 2012; Rosso et al., 2019) and cardiorespiratory performance (Jayakody et al., 2021; Pieruccini-Faria et al., 2020) were also included as confounding factors, as they could influence gait parameters.

An electronic scale (SECA 861, Hamburg, Germany) and a stadiometer (SECA 225, Hamburg, Germany) were used to measure body weight (kg) and height (cm), respectively. Participants were barefoot and wearing light clothes, and both measurements were taken thrice to calculate the average. Then, weight (kg) was divided by height (m^2^) to calculate BMI. The 2-km walking test was used to evaluate cardiorespiratory performance (Oja et al., 1991). Participants walked around a 28 × 15 m rectangular area for 23 laps and 22 m at a maintainable self-selected pace during the whole test. The completion time was recorded in seconds.

### Statistical Analysis

The descriptive characteristics of the study sample are presented as means and standard deviations (*SD*) or percentages, and normality was confirmed for all parameters using the Kolmogorov–Smirnov test. Pearson correlations were conducted between gait variability parameters and between cognitive function variables.

A general linear model approach implemented in SPM12 (London, UK) was conducted for the statistical analyses of imaging data. The associations between each gait variability parameter (i.e., stride length CV, step length CV, step time CV, and gait velocity CV) and GMV were assessed using four separate regressions (one for each gait variability parameter) by voxel-based brain morphometric analysis. Two models were used. Model 1 was adjusted for age, sex, years of education, and BMI as confounders. Model 2 was additionally adjusted for cardiorespiratory performance to determine whether associations between gait variability and GMV are independent of cardiorespiratory performance. The analyses were replicated, including intracranial volume (ICV) as a confounder in all the models.

The GMV clusters were reported after applying AlphaSim spatial extent threshold in the imaging analyses as implemented in Resting-State fMRI Data Analysis Toolkit toolbox (RESTplus; Song et al., 2011). Parameters were defined as follows: cluster connection radius (rmm) = 5 mm and the actual smoothness of the data after model estimation, incorporating a 128,190 voxels gray mask volume. The voxel-level alpha significance (threshold, *p* < .001 uncorrected), along with the appropriate cluster size in each analysis, was indicated in the results. Further Hayasaka corrections were performed to account for the nonisotropic smoothness of structural images and corrected *t* values (Hayasaka et al., 2004). Then, the eigenvalues from the peak coordinates of each significant cluster were extracted, and standardized beta (β) and *p* values were calculated using individual linear regressions using the same confounders.

The associations between gait variability-related GMV (as predictor variables) and cognitive function variables (as dependent variables) were assessed by individual linear regressions using model 2 adjusted by age, sex, years of education, BMI, and cardiorespiratory performance (as confounders). Linear regressions were assessed using the statistical software R studio for Mac, version 4.1.1 (R Foundation for Statistical Computing), with the significance level set at *p* < .05.

Models were tested for sex interactions, and no significant interactions were found (all *p* > .05). Therefore, all analyses were conducted on the entire sample including sex as a confounder.

## Results

### Descriptive Characteristics

Table 1 shows the descriptive characteristics of the study participants. Eighty-seven participants (48 female) were included in the analysis, as four participants were excluded due to insufficient gait data caused by data collection errors. In addition, Pearson correlations between gait variability parameters and between cognitive function variables are shown in Supplementary Figures S2 and S3, respectively.

**Table 1.** Descriptive Characteristics of the Study Sample

Descriptive characteristics	All (*n* = 87)	Male (*n* = 39)	Female (*n* = 48)
Physical characteristics			
Age (years)	71.72 ± 3.99	71.45 ± 3.64	71.93 ± 4.28
Height (cm)	161.02 ± 9.13	168.95 ± 7.11	154.58 ± 4.14
Weight (kg)	74.09 ± 13.20	82.54 ± 11.11	67.22 ± 10.56
Body mass index (kg/m^2^)	28.48 ± 3.91	28.87 ± 3.05	28.16 ± 4.50
Cardiorespiratory performance			
2-km walking test (s)	1,393.73 ± 226.18	1,303.81 ± 180.96	1,466.79 ± 234.46
Education			
Time of education (years)	11.59 ± 4.76	14.10 ± 4.89	9.56 ± 3.56
General cognition			
STICS-m (0-41)	33.99 ± 2.76	34.51 ± 2.49	33.56 ± 2.92
MMSE (0-30)	28.92 ± 1.05	28.85 ± 0.96	28.98 ± 1.12
MoCA (0-30)	25.59 ± 2.14	26.31 ± 1.91	25.00 ± 2.16
Cognitive function			
EF composite (*z*-score)	0.02 ± 0.70	0.32 ± 0.54	-0.23 ± 0.72
Attentional/inhibitory control	0.02 ± 0.68	0.24 ± 0.60	-0.17 ± 0.69
Episodic memory	-0.01 ± 0.60	0.05 ± 0.61	-0.06 ± 0.58
Processing speed	0.01 ± 0.84	0.34 ± 0.65	-0.26 ± 0.89
Visuospatial memory	0.00 ± 1.01	0.17 ± 1.11	-0.15 ± 0.90
Working memory	0.00 ± 0.70	0.12 ± 0.78	-0.10 ± 0.63
Gait variability parameters^a^			
Stride length CV (%)	2.59 ± 0.98	2.34 ± 0.66	2.78 ± 1.15
Step length CV (%)	3.68 ± 1.38	3.15 ± 0.82	4.12 ± 1.58
Step time CV (%)	2.59 ± 0.82	2.26 ± 0.59	2.86 ± 0.89
Gait velocity CV (%)	3.03 ± 1.03	2.83 ± 0.96	3.19 ± 1.06

*Notes*: CV = coefficient of variation; MMSE = Mini-Mental State Examination; MoCA = Montreal Cognitive Assessment; *SD* = standard deviation; STICS-m = Spanish Telephone Interview for Cognitive Status modified. Values are expressed as mean ± *SD*, unless otherwise indicated. Gait variability parameters are expressed as CV ± *SD*.

^a^Lower values of cardiorespiratory performance and gait variability parameters indicate better performance.

### Associations Between Gait Variability Parameters and GMV

**Figure 2.**
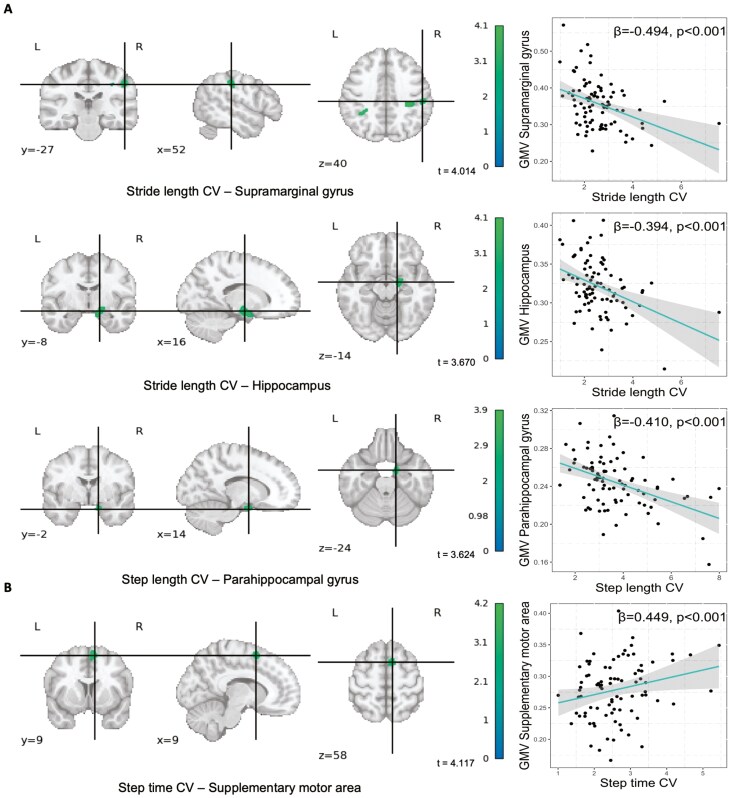
Brain regions showing negative (**A**: stride length and step length) and positive (**B**: step time) associations between gait variability parameters and GMV in cognitively normal older adults (*n* = 87). BMI = body mass index; CV = coefficient of variation; GMV = gray matter volume; L = left; R = right; *x*, *y*, *z* = coordinates of the clusters. Analyses were adjusted by age, sex, years of education, BMI, and cardiorespiratory performance (Model 2). The color bar represents *t* values, with green color indicating stronger association. Plots on the right side of the figure show linear regressions between gait variability parameters and eigenvalues of the GMV clusters.

Table 2 displays the brain regions that showed associations with gait variability parameters. In model 1, greater stride length variability was associated with lower GMV (*p* < .001) in two clusters: supramarginal gyrus (*t* = 4.036, *k* = 173, β = -0.492) and hippocampus (*t* = 3.737, *k* = 468, β = -0.397). In addition, greater step length variability was associated with lower GMV (*p* < .001) in one cluster located in the parahippocampal gyrus (*t* = 3.898, *k* = 140, β = -0.419). Finally, greater step time variability was associated with greater GMV (*p* < .001) in a cluster located in the supplementary motor area (*t* = 4.125, *k* = 278, β = 0.449). In model 2, the previous four clusters remained significant (Figure 2, Table 2). Gait velocity variability did not show any association with GMV in any model (*p* > .05). Analyses replicated, including ICV as a confounder, yielded similar results (see Supplementary Table S3). All results remained consistent when including gait velocity mean as a confounder in the models (data not shown).

**Table 2.** Brain Regions Showing Associations Between Gait Variability Parameters and GMV in Cognitively Normal Older Adults (*n* = 87)

Gait parameter	Brain regions (mm^3^)	*x*	*y*	*z*	Model 1			Model 2		
					Peak *t*	Cluster size (*k*)	β	Peak *t*	Cluster size (*k*)	β
*Negative associations*										
Stride length CV*	Supramarginal gyrus	52	-27	40	4.036	173	-0.492	4.014	179	-0.494
	Hippocampus	16	-8	-14	3.737	468	-0.397	3.670	334	-0.394
Step length CV*	Parahippocampal gyrus	14	-2	-24	3.898	140	-0.419	3.624	76	-0.410
*Positive association*										
Step time CV*	Supplementary motor area	9	9	58	4.125	278	0.449	4.117	274	0.449

*Notes*: CV = coefficient of variation; GMV = gray matter volume; *SD* = standard deviation. Analyses were adjusted by sex, age, years of education, and body mass index (kg/m^2^) in model 1; and additionally adjusted for cardiorespiratory performance in model 2. All contrasts surpassed Hayasaka correction and were thresholded using AlphaSim at *p* < .001 with *k* = 43 voxels for stride length CV, *k* = 51 for step length CV, and *k* = 41 for step time CV in model 1. In model 2, *k* = 46 voxels for stride length CV, *k* = 48 for step length CV, and *k* = 41 for step time CV. Anatomical coordinates (*x*, *y*, *z*) are given in Montreal Neurological Institute (MNI) Atlas space. Beta (β) presented is standardized. Gait variability parameters are expressed as CV ± *SD*.

^*^Lower values of gait CV parameters indicate better performance.

### Associations Between Gait Variability-Related GMV and Cognitive Function

**Figure 3.**
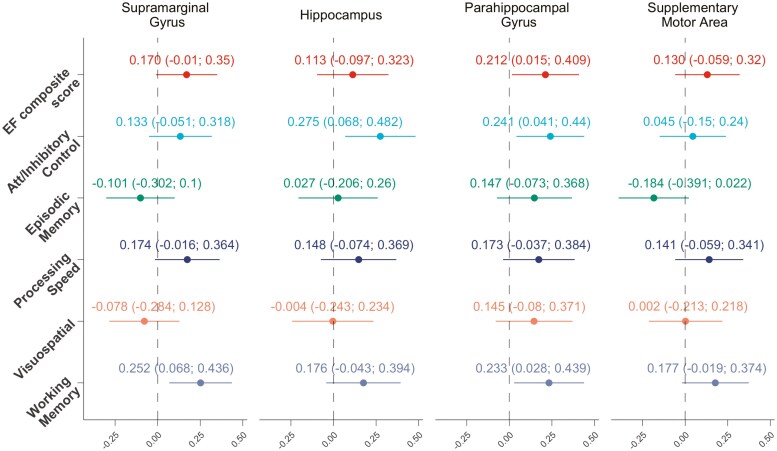
Associations between gait variability-related GMV and cognitive function in cognitively normal older adults (*n* = 87). EF = executive function; GMV = gray matter volume.

Figure 3 shows associations between gait variability-related GMV and cognitive function. As there were no differences between models, model 2, including cardiorespiratory performance, was used for further analyses. Three of the four previously associated peak coordinate clusters presented significant associations with cognitive function. GMV in the supramarginal gyrus was positively associated with working memory (β = 0.252, *p* = .008); GMV in the hippocampus was positively associated with attentional/inhibitory control (β = 0.275, *p* = .010); and GMV in the parahippocampal gyrus was positively associated with EF (β = 0.212, *p* = .035), attentional/inhibitory control (β = 0.241, *p* = .019), and working memory (β = 0.233, *p* = .027). The supplementary motor area region did not show a significant association with cognitive function (*p* > .05).

## Discussion

Our results indicated that most of the gait variability parameters were associated with GMV in cognitively normal older adults. Specifically, greater stride length variability was associated with lower GMV in the supramarginal gyrus and hippocampus regions, and greater step length variability was associated with lower GMV in the parahippocampal gyrus. On the other hand, greater step time variability was associated with greater GMV in the supplementary motor area. No association was found between gait velocity variability and GMV. Additionally, three gait variability-related gray matter clusters showed positive associations with cognitive function, specifically in the supramarginal gyrus, hippocampus, and parahippocampal gyrus. These results may suggest that gait parameters are associated with GMV, with implications for cognitive function in the aging brain.

Although gait variability parameters have been established as better markers for gray matter atrophy and cognitive decline compared to mean gait parameters (Blumen et al., 2019; Callisaya et al., 2014; Kroneberg et al., 2019; Pieruccini-Faria et al., 2020), the literature on this topic is equivocal. Previous studies found associations between gait variability and cognitive functions in normal aging (Hausdorff et al., 2001; Herman et al., 2010; Ijmker & Lamoth, 2012; Párraga-Montilla et al., 2021; Van Iersel et al., 2008). However, there is only one previous study that assessed the associations between gait variability parameters, GMV, and cognitive function using similar methods as ours in a similar population (Cullen et al., 2018). Notably, this study is not fully comparable with ours, as they did not identify specific regions of interest but rather covariance pattern networks, and they measured EF using different cognitive tests than those used in our study (Ali et al., 2022; Rosso et al., 2014). Likewise, the associations we found between gait variability-related GMV and cognitive function complement the compelling evidence linking gait variability to gray matter atrophy and cognitive impairment (Callisaya et al., 2013; Lindh-Rengifo et al., 2022). Our study suggests that stride length variability, step length variability, and step time variability may be three gait parameters detecting gait impairments earlier through aging and neurodegenerative decline (Párraga-Montilla et al., 2021).

The lower GMV in the supramarginal gyrus, identified in our study as an association with greater stride length variability, has been previously related to the processing of sensory, proprioceptive, auditory, visual, and somatosensory information (DiSalvio et al., 2020). These associations might indicate disruptions in sensory processes, potentially having an impact on gait stability and coordination. Furthermore, the lower GMV in the hippocampus and parahippocampal gyrus, identified in our study as an association with greater stride length variability and greater step length variability, respectively, have been related to spatial memory, memory retrieval, and facial expression recognition (Jayakody et al., 2021). This suggests a link between impairments in these processes and alterations in the gait pattern, and the control of gait and the rhythmic step mechanism during normal aging (Beauchet et al., 2015). These findings may imply that gait control plays a crucial role in cognitive processes related to somatosensory perception, spatial awareness, and memory. Also, atrophy in these brain regions may contribute to gait impairment and issues with gait stability. Future studies with a longitudinal design should test the bidirectionality of these associations.

Conversely, and inconsistent with our predictions, we found that greater step time variability was associated with greater GMV in the supplementary motor area. A previous study in healthy older adults did not identify any association between step time variability and gray matter covariance patterns, although this study examined gray matter networks rather than individual brain regions (Jayakody et al., 2021). The supplementary motor area is related to planning complex sequences of movements and coordinating bilateral movements. The observed relationship between step time variability and this brain region may be attributed to mechanisms in which potential markers of reserve functions could act to mitigate the deterioration of other brain regions. A plausible explanation is that deterioration in specific brain regions may trigger pathways in which years of compensatory neural effects might lead to an increase in volume in other regions (Rosso et al., 2014). This mechanism, increasing neural activity, could serve as a potential compensation strategy, enabling individuals to overcome difficulties in maintaining consistent step times during gait (Lipat et al., 2022). Furthermore, a previous study stated that step time consistency may largely be under spinal rather than supraspinal locomotor control (Dietz, 2003).

There are some additional explanations beyond compensatory mechanisms for the contrasting associations between different gait variability parameters and specific brain regions (Rosso et al., 2014). First, the differential impact of aging is a factor, as brain regions might respond differently to aging. Some areas might experience atrophy (resulting in negative associations), while others undergo plastic changes, enhancing their function (resulting in positive associations; Beauchet et al., 2009). Second, the multifaceted nature of gait—encompassing a complex interplay of sensory, motor, and cognitive processes—may help explain the divergent neural associations observed across different gait parameters (Chen et al., 2020; Rosso et al., 2014). Different aspects of gait variability (stride length, step length, and step time) might involve distinct neural circuits, explaining the diverse associations. Finally, individual variability plays a role, as each person’s neural architecture and gait patterns are unique. Variability in associations could stem from individual differences, including genetic factors, lifestyle, and overall health.

We did not identify any association between gait velocity variability and GMV, even though decreased gait velocity has been widely associated with brain atrophy and cognitive decline (Callisaya et al., 2013, 2014). This finding is significant because it may suggest that while overall gait velocity may be an indicator of brain health as reported in previous studies, gait velocity variability does not appear to correlate with GMV in the present study. There is limited research on the gait impairment linked to morphological changes in brain volume in individuals experiencing cognitive decline, although evidence supports that gait velocity has been strongly linked to cognitive performance (Ali et al., 2022). By utilizing gait parameters as a marker of early brain decline that might have implications for downstream cognitive performance, we may start to promote targeted strategies. These strategies could potentially include lifestyle, behavioral changes, and training interventions to mitigate cognitive decline prior to the onset of clinical symptoms (Rosso et al., 2014).

Importantly, our study revealed a positive association between a gait variability-related GMV cluster in the parahippocampal gyrus and EF (Jayakody et al., 2021). Although the parahippocampal gyrus is traditionally linked to memory and spatial navigation, emerging evidence suggests its involvement in EF through its roles in attentional control, inhibition, and retrieving information necessary for decision-making and planning, particularly in aging populations (Beauchet et al., 2015; Jayakody et al., 2021). Although our findings demonstrate an association between the parahippocampal cluster and EF, the directionality of that relationship remains unclear and warrants further investigation. As both gait and cognition are behaviors resulting from complex neurobiological processes (Zimmerman et al., 2009), it is plausible that age- or pathology-related GMV atrophy may contribute to changes in gait variability, which could in turn affect EF. Conversely, cognitive impairment may indirectly influence neurodegenerative progression by reducing cognitive reserve through diminished physical activity, social engagement, or other lifestyle factors (Erickson et al., 2022). Future studies should explore theoretical models, including potential mediation or moderation effects, to better understand how GMV in that region may bridge gait variability and cognition (Tian et al., 2017). Additionally, we cannot exclude the possibility that these associations are influenced by unmeasured variables, such as lifestyle or health factors not included in our models. Longitudinal studies will be essential to clarify these mechanisms and establish whether gait variability serves as a marker, mediator, or consequence of neurodegenerative and cognitive processes (Rosso et al., 2014). Collaboration and harmonization of protocols are needed to facilitate easier comparison of results across studies.

Our findings further support the link between gait variability-related GMV and specific cognitive domains, reinforcing the role of these brain structures in cognitive aging. We observed that GMV in the supramarginal and parahippocampal gyri was positively associated with working memory, while GMV in the hippocampus and parahippocampal gyrus showed positive associations with attentional/inhibitory control. These associations are partially consistent with the established functional roles of these regions. The supramarginal gyrus has been implicated in sensory integration and working memory processes, which are critical for cognitive flexibility and EF (Annweiler et al., 2013). Likewise, the hippocampus and parahippocampal gyrus, traditionally associated with memory and spatial navigation, also play a key role in attentional control and inhibitory processing, functions that are essential for goal-directed behavior (Beauchet et al., 2015; Jayakody et al., 2021). Given that attentional/inhibitory control and working memory were the cognitive domains most strongly correlated with the EF composite score in our study (*r* ≥ 0.79), it is reasonable that they exhibited the strongest associations with gait variability-related GMV. In contrast, the supplementary motor area did not show significant associations with EF or cognitive domains, suggesting that its role in gait variability may be more related to motor control and compensatory mechanisms rather than direct cognitive processing (Lipat et al., 2022). These findings highlight the importance of considering domain-specific cognitive associations (Oberlin et al., 2024) when investigating the interplay between gait variability, brain structure, and cognitive function in aging populations. Future research should aim to clarify the potential causal pathways linking gait variability, GMV, and cognition, as well as explore whether gait variability can serve as an early marker of cognitive decline in neurodegenerative processes.

The current study had limitations. For instance, the cross-sectional design limits causal inferences and hinders the identification of the temporal direction of the associations, preventing conclusions about whether changes in gait or brain occur first over time. Our analyses were conducted with gait variability as the independent variable and GMV as the dependent variable following and based on previous studies, and we did not examine the inverse direction due to the complexity introduced by our whole-brain approach. Future longitudinal studies are necessary to explore bidirectional relationships and determine whether gait variability reflects underlying neurodegenerative processes or contributes to them, further clarifying its role as a potential marker of aging and cognitive decline. Furthermore, due to the cognitively normal status of our population, our findings might not apply to older adults with cognitive impairment or dementia. As our data were collected in an RCT, the sample may not fully represent the broader population due to potential sampling biases, such as inclusion criteria, willingness to participate, and proximity to research centers. Finally, leg length was not collected during our study, so we were unable to include it as a confounder. Our study also has several strengths, one of which is the inclusion of a relatively large sample of cognitively normal older adults. We measured a series of standardized gait variability markers with the potential capacity to predict brain atrophy. Additionally, we included the implications of gait variability-related GMV on cognitive function, enabling us to link brain structure with behavioral measures.

## Implications

Our results revealed significant associations between gait variability parameters and specific brain regions: (i) greater stride length variability was associated with lower GMV in the supramarginal gyrus and hippocampus; (ii) greater step length was associated with lower GMV in the parahippocampal gyrus; and (iii) greater step time variability was associated with greater GMV in the supplementary motor area. These associations with GMV in the supramarginal gyrus, hippocampus, and parahippocampal gyrus were also associated with cognitive function, suggesting that impaired gait regulation is related to brain structure, with implications for cognitive abilities in aging individuals. These findings propose that gait impairments during aging play a significant role in specific cognitive processes and may potentially contribute to brain atrophy. Understanding these associations is essential for early dementia detection and sheds light on the complex interplay between physical function, brain health, and cognitive function during aging. Notably, gait variability may be a simple measure that could potentially serve as an indicator of neurocognitive decline in healthy aging and neurodegenerative disorders. However, longitudinal studies tracking individuals over time are warranted to examine the bidirectionality between gait performance, brain structure, and cognitive function.

## Supplementary Material

igaf045_suppl_Supplementary_Materials

## Data Availability

Data from this study cannot be made available to other researchers for reasons of protecting the anonymity of the participants and because they did not provide consent for their raw data to be shared publicly. Analytic methods or materials are available to other researchers for replication purposes. Data are available in the GitHub open repository (https://github.com/aguedaprojectugr/gait_whole_brain_cognition). Our study uses secondary data that was not preregistered.
