# Supplementary material for: Gait Variability Is Associated With Gray Matter Volumes Implicated in Cognitive Function: A Cross-Sectional Analysis From the AGUEDA Trial
Source: Innov Aging. 2025 May 6;9(6):igaf045. doi: 10.1093/geroni/igaf045 (PMC12246678; doi:10.1093/geroni/igaf045)
Supplement: igaf045_suppl_Supplementary_Materials [file igaf045_suppl_supplementary_materials.docx]

***Innovation in Aging* Supplementary Material:** Martín-Fuentes et al. **Gait variability is associated with gray matter volumes implicated in cognitive function: A cross-sectional analysis from the AGUEDA trial.**

**Supplementary Table 1.** Descriptive characteristics of gait parameters.

|  | | Description | All  (n=87) | Male  (n=39) | Female  (n=48) |
| --- | --- | --- | --- | --- | --- |
| **Gait parameters** | |  |  |  |  |
| Stride length (cm) | | Distance between heel points of two consecutive footsteps of the same foot. | 143.68 ± 18.41 | 155.75 ± 16.07 | 133.88 ± 13.86 |
| Step length (cm) | | Distance between the heel points of one foot and the other foot. | 71.73 ± 9.16 | 77.65 ± 8.05 | 66.92 ± 6.96 |
| Step time (s) | | Duration from the contact of one foot to the contact of the opposite foot. | 0.47 ± 0.04 | 0.48 ± 0.04 | 0.47 ± 0.03 |
| Gait velocity (m/s) | | Mean velocity referred to a step. | 1.53 ± 0.23 | 1.63 ± 0.23 | 1.45 ± 0.20 |
|  | Note: Values are expressed as mean ± SD. | | |  |  |

**Supplementary Table 2.** Tests included in the cognitive domains.

| **Cognitive Domain** | **Tests Included** |
| --- | --- |
| 1. Attentional/inhibitory control | Flanker  Stroop Test (incongruent trial)  Dimensional Change Card Sort Test  Trail Making Test, Part B |
| 1. Episodic memory | MoCA delayed recall  Rey Auditory Verbal Learning Test  Rey-Osterrieth Complex Figure Test (ROFT)  Picture Sequence Memory Test |
| 1. Processing speed | Digit Symbol Substitution Test  Trail Making Test, Part A |
| 1. Visuospatial processing | Wechsler Adult Intelligence Scale with Matrix Reasoning  Block Design and MoCA Clock Draw |
| 1. Working memory | N-Back Working Memory Test  Spatial Working Memory Test  List Sorting Working Memory Test |

**Supplementary Table 3.** Brain regions showing associations between gait variability parameters and GMV in cognitively normal older adults (n=87). Models additionally adjusted by intracranial volume.

|  |  |  | |  | |  | *Model 1* | | | | *Model 2* | | | |
| --- | --- | --- | --- | --- | --- | --- | --- | --- | --- | --- | --- | --- | --- | --- |
| **Gait**  **parameter** | **Brain Regions (mm^3^)** | **X** | **Y** | | **Z** | | **Peak t** | **Cluster size (k)** | ***β*** | **Peak t** | | **Cluster size (k)** | ***β*** |  |
| **Negative associations** |  |  |  | |  | |  |  |  |  | |  |  |  |
| Stride length CV | Supramarginal Gyrus | 52 | ⎼27 | | 40 | | 4.060 | 192 | ⎼0.475 | 4.075 | | 224 | ⎼0.480 |  |
|  | Hippocampus | 15 | ⎼9 | | ⎼16 | | 3.803 | 671 | ⎼0.381 | 3.765 | | 565 | ⎼0.382 |  |
| Step length CV | Parahippocampal Gyrus | 14 | 0 | | ⎼22 | | 4.343 | 521 | ⎼0.434 | 4.154 | | 384 | ⎼0.436 |  |
| **Positive association** |  |  |  | |  | |  |  |  |  | |  |  |  |
| Step time CV | Supplementary Motor Area | 9 | 9 | | 58 | | 4.230 | 307 | 0.432 | 4.212 | | 302 | 0.432 |  |
|  |  |  |  | |  | |  |  |  |  | |  |  |  |

Note: CV = coefficient of variation. Analyses were adjusted by sex, age, years of education, body mass index (kg/m^2^), and intracranial volume in model 1; and additionally adjusted for cardiorespiratory performance in model 2. All contrasts surpassed Hayasaka correction and were thresholded using AlphaSim at p < 0.001 with k = 48 voxels for stride length CV, k = 52 for step length CV, and k = 40 for step time CV in model 1. In model 2, k = 46 voxels for stride length CV, k = 50 for step length CV, and k = 41 for step time CV. Anatomical coordinates (X, Y, Z) are given in Montreal Neurological Institute (MNI) Atlas space. Beta (β) presented is standardized. Gait variability parameters are expressed as CV ± SD. *Lower values of gait CV parameters indicate better performance.

**
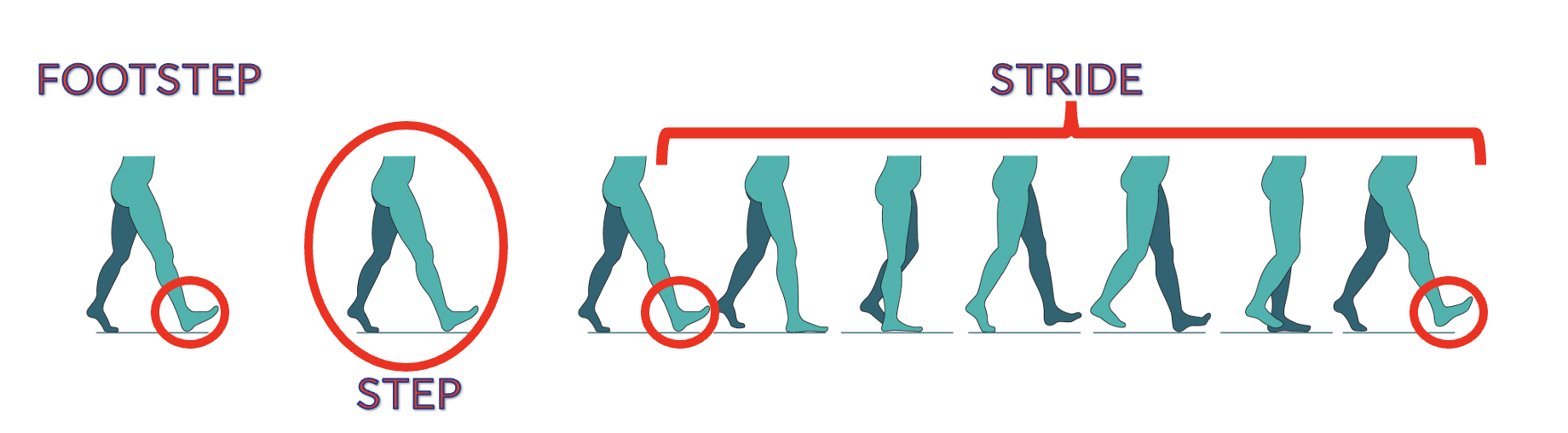
**

**Supplementary Figure 1.** Graphical description of the parameters.

**
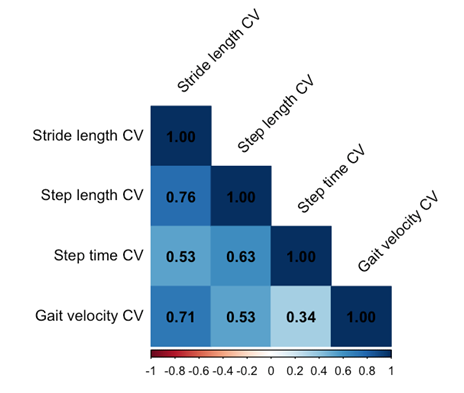
**

**Supplementary Figure 2.** Pearson correlations between gait variability parameters. Color bar represents the strength of the correlation as r values. The darker blue the color, the stronger the positive correlation between variables. CV: coefficient of variation. All p < 0.05.

**
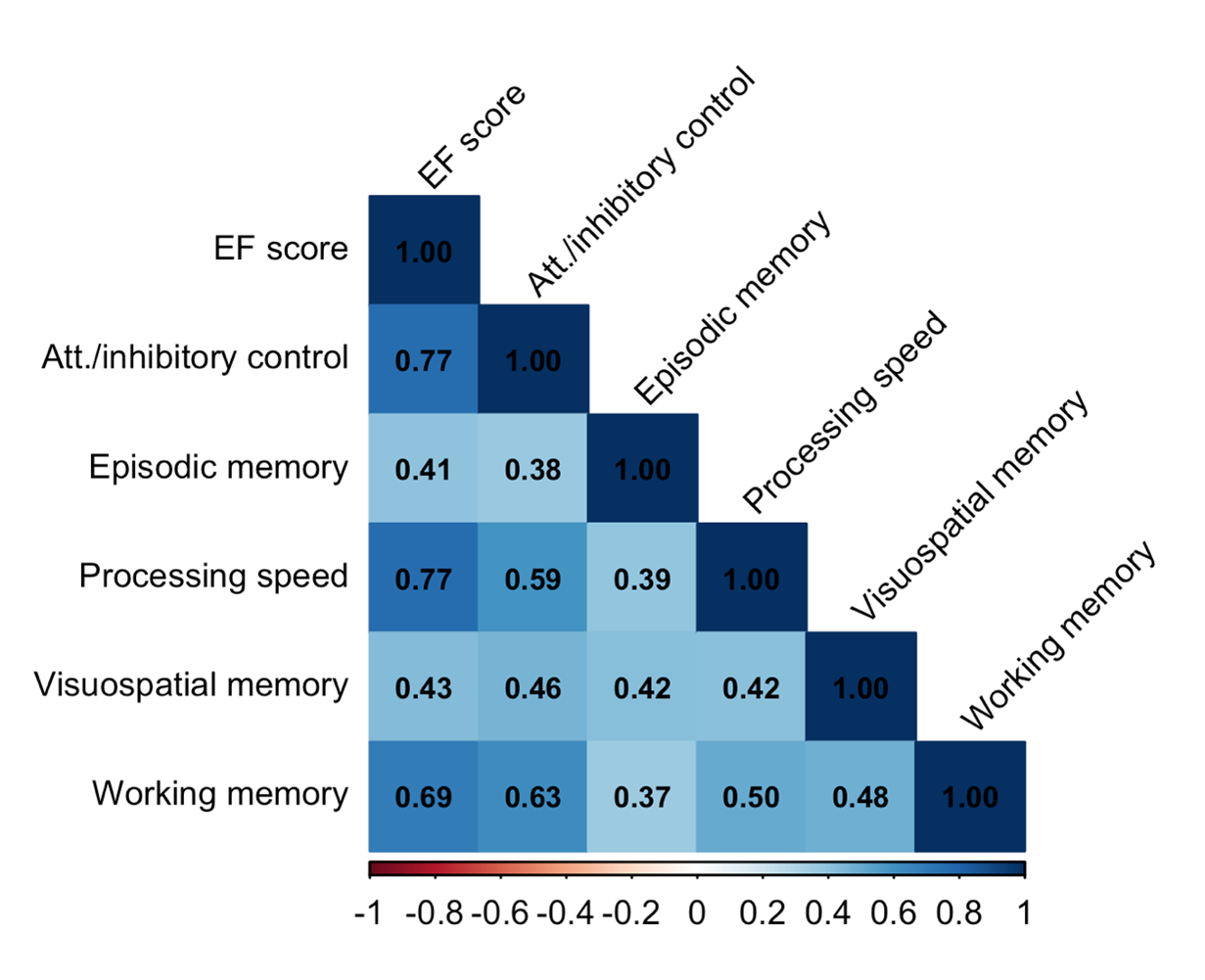
**

**Supplementary Figure 3.** Pearson correlations between cognitive function variables. Color bar represents the strength of the correlation as r values. The darker blue the color, the stronger the positive correlation between variables. CV: coefficient of variation. All p < 0.05.
